# Discoveries Interview: Professor Greg Gibson on the genomics revolution

**DOI:** 10.15190/d.2015.44

**Published:** 2015-12-31

**Keywords:** interview, discovery, Gregory Gibson, cryptic genetic variation, Center for Integrative Genomics, Emory University, Georgia Institute of Technology, discoveries journals

**Figure 1:**
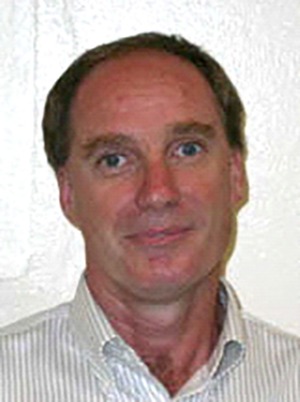
Professor Greg Gibson

**Dr. Greg Gibson** is a Professor in the School of Biology at the Georgia Institute of Technology, Atlanta, Georgia, USA, where he directs the Center for Integrative Genomics.

Prof. Gibson is a human evolutionary quantitative geneticist, known for his work on the contributions of cryptic genetic variation and canalization on evolution and disease^1^. He trained as a Drosophila developmental geneticist and spent the first 15 years of his independent career helping to applying genomic methodologies to the analysis of complex traits in Drosophila, and was elected a Fellow of the AAAS for these efforts. For the past 10 years he has refocused on human genetics, with particular interests in gene expression profiling, genotype-by-environment interactions, and integrative genomics approaches to personalized medicine. He received his BSc from the University of Sydney in Australia, and PhD from the University of Basel in Switzerland, where he worked under the supervision of the late Prof. Walter Gehring. After Post-Docs at Stanford University with David Hogness, and briefly with Cathy Laurie at Duke University where he received training in evolutionary genetics, he was an Assistant Professor at the University of Michigan, and then Associate and Full Professor at NC State University. He has been at Georgia Tech since 2009. Professor Gibson is the author of two text-books published by Sinauer Associates (A Primer of Genome Science, with Spencer Muse, first edition in 2001, and A Primer of Human Genetics, first published in 2015), as well as a popular science book, It Takes a Genome (FT Press, 2009). He serves as section editor for Natural Variation at PLoS Genetics. His monthly blog is at: http://www.genomestake.blogspot.com/

## 1. Can you define in simple words what genomics and integrative genomics are and why are they important?

Genomics is the study of the structure and function of the genetic material. Thirty years ago, molecular genetics was performed one gene at a time, which was fine for developmental and Mendelian geneticists. Those interested in quantitative genetics (that is, complex traits influenced by many genes and the environment) and/or evolutionary genetics did not really have the tools to do more than theorize. Then as DNA sequencing technology brought the capacity to sequence whole genomes, and computer science enabled bioinformatics, the discipline of genomics emerged. In parallel, microarray technology allowed us to quantify gene expression genome-wide, and the tools of proteomics and metabolomics and now microbiome analysis have progressed, so that we can now study not just the genome, but also all of their products. Putting all of the pieces together is called integrative genomics. The term systems biology can be used inter-changeably, although I like to think of integrative genomics as the application of statistical methods to mine complex omic datasets, whereas systems biology is the epistemological strategy of using genomics to generate and test hypotheses in an iterative manner^2^. Together they open the black box between genotype and phenotype and give us a much richer picture of the complexity of molecular mechanisms than can be obtained by only studying the genome.

## 2. How genomics field evolved over the time?

It is a young field still, but seems to be quite mature in so far as the domains of research (genomics, transcriptomics, epigenomics, proteomics, metabolomics, phenomics) each have their own lore. Yet integrative genomics also seems to go through fundamental shifts every five years. I hesitate to say revolutions, since you can pretty much see how the trends change in an incremental manner – but the increments are accumulating so rapidly that what we can do today was barely conceivable a quarter century ago. Who would have thought that 15 years after the first draft human genome was published, the whole genome sequences of a third of a country (Iceland) would be generated!^3^ Much of this is driven by technological advances, particularly DNA sequencing and data processing capacity, but equally important has been the emergence of large collaborative networks. This is particularly apparent in human genetics where a largish cohort of 1000 people is now a footnote to an international consortium of a quarter million subjects.

## 3. Why next-generation sequencing is important for our understanding, detection and treatment of various diseases such as cancer?

When we talk about personalized medicine, there are two broad streams that can be referred to as precision medicine and predictive health^4^. Broadly speaking, precision medicine is about diagnosing the molecular cause of a condition, whereas predictive health is about trying to predict and prevent the onset of disease. At least today, most precision medicine deals with rare variants or mutations that are necessary (though not necessarily sufficient) for disease. It is already being applied in the clinic for pediatric abnormalities^5^and for personalized cancer diagnosis and therapy^6^. Up until a couple of years ago, clinical geneticists were limited to targeted sequencing of a few dozen genes that were good candidates for any particular case, but these often came up empty at great expense. Now whole exome sequencing (and soon, whole genome) will cover all of the genes in the genome for around a thousand dollars, and typically find a very strong candidate mutation a third of the time. In cancer, integration of genome sequencing with methylation and transcriptome data is identifying drivers of specific tumors or mechanisms of drug resistance, suggesting most likely therapeutics. We’re quite a way conceptually from being able to do similar things for the common diseases that affect tens of millions of people (diabetes, asthma, arthritis, dementia, depression), but the eventual goal is to be able to tailor therapy and/or prevention to individual circumstances.

## 4. What will the field look like in 5-10 years?

Right now, there is a tension between the scale of discovery genomics soon to be performed on datasets of millions of people, and the desire to translate the findings at the level of N=1. I imagine that sequencing technology will continue to improve to the point where a whole genome and transcriptome and microbiome can be generated for a few hundred dollars in a few hours, and no doubt nano-scale proteomes and metabolomes will become available as well. We will have lists of which genes and metabolites are most often contributing to causation, and massive databases of drug response will provide the evidence physicians need to treat given a molecular diagnosis, but I think this is decades (plural) in the future. New bioinformatics strategies will eventually allow us to move beyond statistical models of risk and to incorporate the genomic findings into mathematical models of cellular and organismal physiology. So, over the next 10 years I see bigger and bigger scale, more and more sophisticated bioinformatics, and a shift from discovery to translational medicine. Of course, in parallel integrative genomics will also continue to reshape evolutionary and organismal biology and agriculture.

## 5. What advices do you have for young scientists?

Someone once pointed out that there are three hard things about being a scientist: asking a good question, doing the research to address it, and going public with your findings. Usually your post-doc is the time where you begin to ask your own questions, and then setting up your own group it is essential to establish your own identity. This is hard now that so much (genomic) science is collaborative and team-oriented, but I still think it is always best to express your individuality by original and passionate pursuit of your own ideas. Then when it comes to publishing or speaking in public, you need a thick skin, since anything new is always going to be subject to criticism. The rewards though are enormous, outside the formal channels of review, you will develop a community of friends and colleagues all over the world who appreciate and embrace discovery and creativity.

## 6. In your opinion, what are the most challenging, promising and/or the most rewarding areas of research?

Oddly enough, I think the biggest challenges for integrative genomics are simultaneously establishing relevance and meeting expectations. By relevance, I mean showing that the integrative approach really does improve diagnosis or enhance predictive health. The dominant paradigm currently is DNA-sequence based genetics on the supposition that specific mutations are sufficient to cause disease or drive cancer. But most of the time it is much more complex – for example, we are just beginning to see how a person’s microbiome modifies the immune and nervous systems. The potential of systems biology is to extend our understanding from a minority to the majority of cases – but therein lays the burden of expectation^7^. Actually, as a biologist who uses the tools of integrative genomics, I would like to conclude with the comment that perhaps the most rewarding part of all of this research is that integrative links reductionist to holistic, and reminds us that molecular explanations are only a part of the story.
